# ChEMBL web services: streamlining access to drug discovery data and utilities

**DOI:** 10.1093/nar/gkv352

**Published:** 2015-04-16

**Authors:** Mark Davies, Michał Nowotka, George Papadatos, Nathan Dedman, Anna Gaulton, Francis Atkinson, Louisa Bellis, John P. Overington

**Affiliations:** European Molecular Biology Laboratory - European Bioinformatics Institute, Wellcome Trust Genome Campus, Hinxton, Cambridgeshire CB10 1SD, UK

## Abstract

ChEMBL is now a well-established resource in the fields of drug discovery and medicinal chemistry research. The ChEMBL database curates and stores standardized bioactivity, molecule, target and drug data extracted from multiple sources, including the primary medicinal chemistry literature. Programmatic access to ChEMBL data has been improved by a recent update to the ChEMBL web services (version 2.0.x, https://www.ebi.ac.uk/chembl/api/data/docs), which exposes significantly more data from the underlying database and introduces new functionality. To complement the data-focused services, a utility service (version 1.0.x, https://www.ebi.ac.uk/chembl/api/utils/docs), which provides RESTful access to commonly used cheminformatics methods, has also been concurrently developed. The ChEMBL web services can be used together or independently to build applications and data processing workflows relevant to drug discovery and chemical biology.

## INTRODUCTION

The ChEMBL database (1,2) is the largest primary Open Data source of manually extracted and curated Structure Activity Relationship data from the medicinal chemistry literature. The primary relationship captured in the ChEMBL database is the association between a ligand and a biological target in the form of an experimentally measured activity end-point, e.g. half maximal inhibitory concentration (IC50). The properties of a successful drug though do not solely derive from a single potency against a specific target, thus the ChEMBL database also contains many additional bioassays, such as efficacy in functional assays, ADME and toxicity end-points and physicochemical properties. Further curation and standardisation of data are carried out and additional calculated properties and annotations are added, e.g. names and synonyms, target information, calculated properties and structure representations, and drug mechanism of action.

ChEMBL data is made available in multiple formats. For in-house integration purposes, it is possible to download relational database exports, flat-file representations and the myChEMBL virtual machine (3,4). Online access is provided through the ChEMBL web interface (https://www.ebi.ac.uk/chembl/), the EBI-RDF platform (5) and the ChEMBL web services. The data in ChEMBL is licensed under CC-BY-SA (https://creativecommons.org/licenses/by-sa/3.0/). Additionally ChEMBL data is also available via other resources that integrate ChEMBL data, such as BindingDB (6) and PubChem BioAssay (7,8). The ChEMBL web services are built using a RESTful architecture and provide users with programmatic access to the data. A recent update to the web services exposes more data from the underlying ChEMBL database and also provides new functionality, both of which significantly improve data integration opportunities.

## CHEMBL WEB SERVICE UPDATE

The ChEMBL web services were first released in 2011. Since this first unpublished web service release, the content and data model of the underlying ChEMBL database have evolved significantly and these changes have not been propagated through to the respective web services. In this publication we describe an updated set of web services, which expand access to ChEMBL data and also include a number of new features, such as advanced data filtering, ordering and paging capabilities. A comparison between the updated ChEMBL web services and the previous version is presented in Supplementary Table S1.

## CHEMBL WEB SERVICE SOFTWARE ARCHITECTURE

The ChEMBL database (release 20) consists of 63 tables. The tables and the relationships between them can be seen in the ChEMBL release schema diagram (ftp://ftp.ebi.ac.uk/pub/databases/chembl/ChEMBLdb/releases/chembl_20/chembl_20_schema.png). To assist with Create, Read, Update and Delete operations on the database, a detailed model has been created of the database using Object Relational Mapping (ORM) technique. An ORM-based model maps each table within a database schema to a software class and the columns and relationships between tables are mapped to class attributes and methods respectively. One advantage of using an ORM-based model is that it avoids the use of raw SQL when interacting with the database, which is often a source of hard to detect errors and vulnerabilities in code bases. The ChEMBL ORM model is also database agnostic, which allows the same code to be executed on different database engines. The ORM model built for the ChEMBL database provides developers with a convenient way to access the data stored in underlying database. In the case of the ChEMBL ORM model, it did not make sense to expose it directly, as this would require the user to perform multiple http requests in order to handle the complex database relationships, which can increase application complexity and have a negative impact on the speed. Instead, a set of RESTful web service resources have been built on top ChEMBL ORM model. In the case of the ChEMBL web services, ‘Molecule’ would be an example of a web service resource. It is important to design each resource with a data granularity level that provides efficient access to the underlying data. For example, if a user is interested in one or more molecules and their associated molecular properties (e.g. molecular weight) and structural representation (e.g. InChI string), the user would need to make multiple references to related objects, retrieving data from the molecule_dictionary, molecule_properties and compound_structure tables. It would be more convenient if this data was aggregated under a single Molecule resource. The approach of aggregating data about specific domains, e.g. Assay, Molecule, Activity and Target into a single resource, has been used to build the ChEMBL web services. Table 1 provides the full list of ChEMBL data-focused web services.

**Table 1.** ChEMBL web service resources

Example	Description	Name
https://www.ebi.ac.uk/chembl/api/data/activity	Activity values recorded in an Assay	Activity
https://www.ebi.ac.uk/chembl/api/data/assay	Assay details as reported in source Document/Dataset	Assay
https://www.ebi.ac.uk/chembl/api/data/atc_class	WHO ATC Classification for drugs	ATC
https://www.ebi.ac.uk/chembl/api/data/binding_site	Target binding site definition	BindingSite
https://www.ebi.ac.uk/chembl/api/data/biotherapeutic	Biotherapeutic molecules	Biotherapeutic
https://www.ebi.ac.uk/chembl/api/data/cell_line	Cell line information	CellLine
https://www.ebi.ac.uk/chembl/api/data/chembl_id_lookup	Look up ChEMBL Id entity type	ChEMBL-IdLookup
https://www.ebi.ac.uk/chembl/api/data/document	Document/Dataset from which Assays have been extracted	Document
https://www.ebi.ac.uk/chembl/api/data/mechanism	Mechanism of action information for FDA-approved drugs	Mechanism
https://www.ebi.ac.uk/chembl/api/data/molecule	Molecule/biotherapeutics information	Molecule
https://www.ebi.ac.uk/chembl/api/data/molecule_form	Relationships between molecule parents and salts	MoleculeForm
https://www.ebi.ac.uk/chembl/api/data/target	Targets (protein and non-protein) defined in Assay	Target
https://www.ebi.ac.uk/chembl/api/data/target_component	Target sequence information (A Target may have 1 or more sequences)	Target-Component
https://www.ebi.ac.uk/chembl/api/data/image/CHEMBL1	Graphical (png, svg, json) representation of Molecule	Image
https://www.ebi.ac.uk/chembl/api/data/protein_class	Protein family classification of TargetComponents	Protein- Classification
https://www.ebi.ac.uk/chembl/api/data/substructure/CN%28CCCN%29c1cccc2ccccc12	Molecule substructure search	Substructure
https://www.ebi.ac.uk/chembl/api/data/similarity/CC%28=O%29Oc1ccccc1C%28=O%29O/70	Molecule similarity search	Similarity
https://www.ebi.ac.uk/chembl/api/data/source	Document/Dataset source	Source

## APPLICATIONS AND EXAMPLES

### Web service documentation

**Figure 1.**
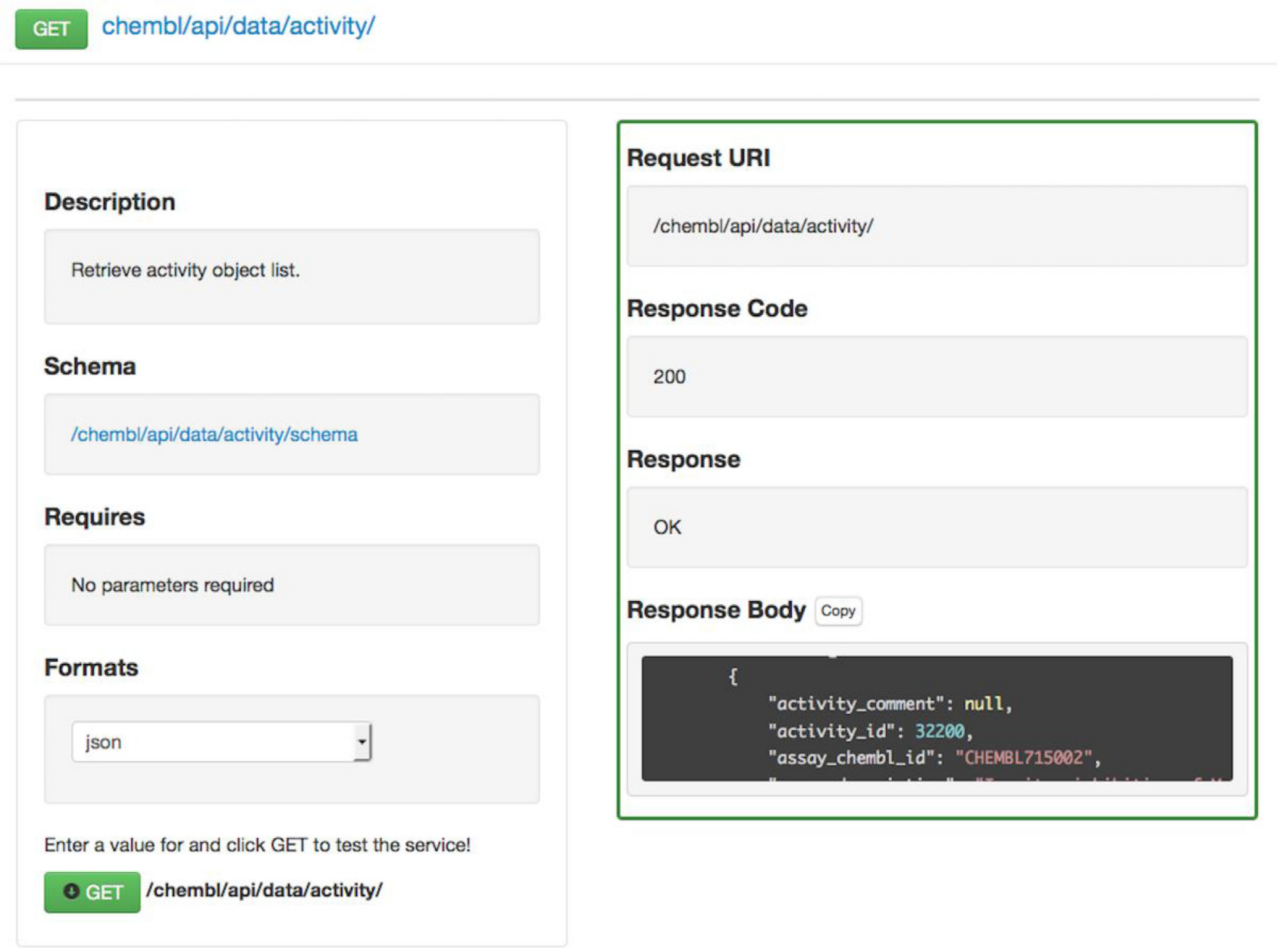
Interactive online SPORE documentation for the Activity resource.

ChEMBL contains many different entity types, which include molecules, bioactivity data points, assay protocols, documents, proteins, cell lines and organisms. To facilitate the discovering and exploring of the web service resources, interactive documentation has been made available online (https://www.ebi.ac.uk/chembl/api/data/docs). The documentation page lists all of the web service resources, which when clicked, will expand to display a description, required parameters and supported response formats. The documentation page can also be used to submit an example request to a resource and view the corresponding response. Figure 1 shows the documentation for the Activity resource, where request details are displayed on the left and response details on the right, making it easy for a user to view and copy the data returned by the web service call. The documentation has been written in accordance with the SPORE specification (https://github.com/SPORE/specifications) and the meta-description used to generate the web service documentation is available online https://www.ebi.ac.uk/chembl/api/data/spore. The online meta-description of the ChEMBL web services allows for the documentation to be generated automatically using the ‘chembl_tastypie_docs’ python package (https://github.com/chembl/tastypie_spore_docs). The ‘chembl_tastypie_docs’ package can be used to automatically document any RESTful API generated using the Python Tastypie library. Additional documentation and example queries using the ChEMBL web services are available online https://www.ebi.ac.uk/chembl/ws.

### Traversing the web services

**Figure 2.**
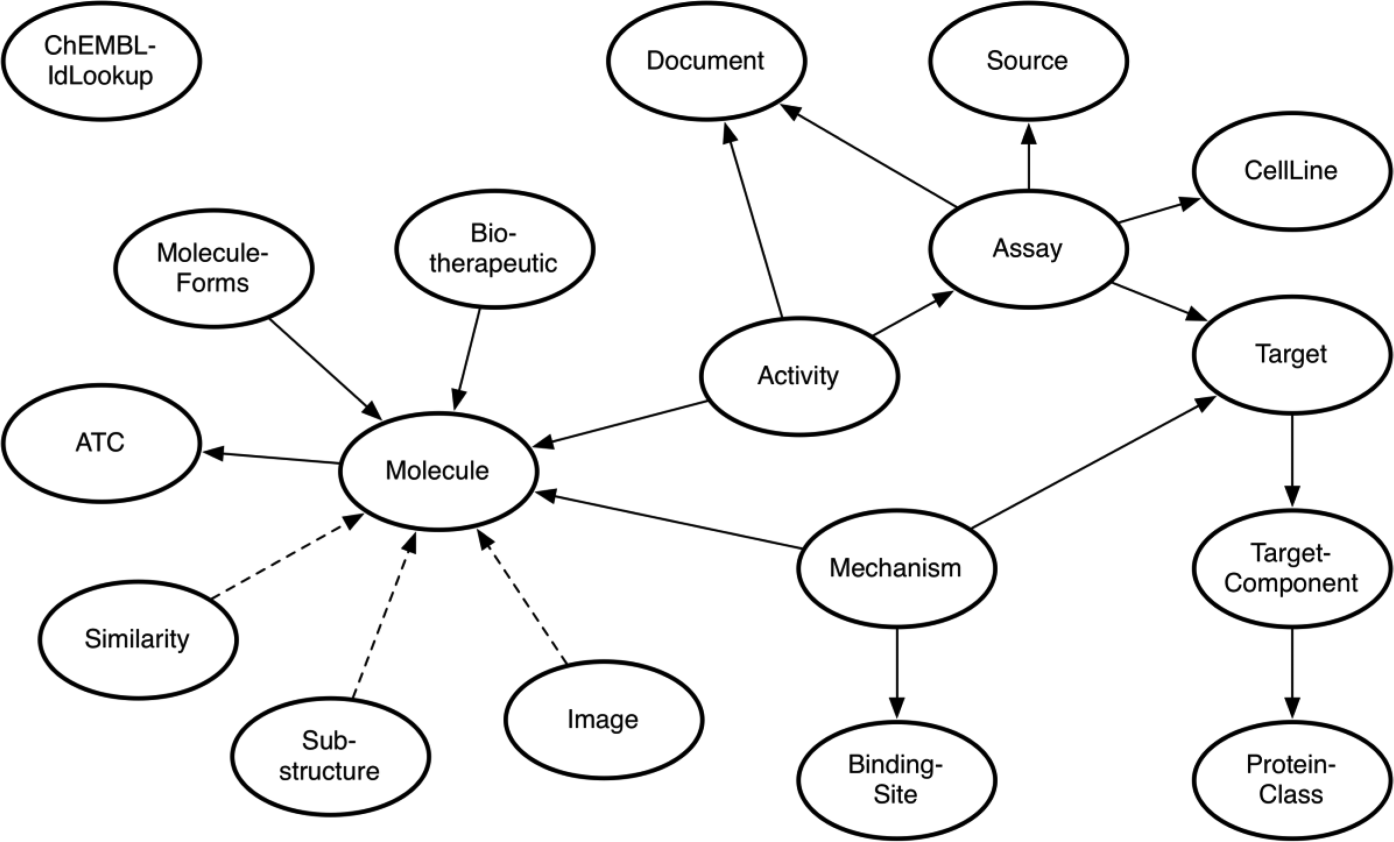
ChEMBL web service schema diagram. The oval shapes represent ChEMBL web service resources and the line between two resources indicates that they share a common attribute. The arrow direction shows where the primary information about a resource type can be found. A dashed line indicates the relationship between two resources behaves differently. For example the Image resource provides a graphical based representation of a Molecule.

When a user interacts with a relational database, the initial point of reference will be the schema diagram. The schema diagram describes the data content of each table and the relationships between the tables. This allows a user to build an SQL query that will retrieve a specific subset of data from the database. The ChEMBL web services allow users to retrieve and filter all data for a specific resource, for example to retrieve all targets from the ChEMBL web services the following URL can be called https://www.ebi.ac.uk/chembl/api/data/target. To ask a more complicated question such as *return the protein targets, that interact with drugs classified as being used in the treatment of diabetes*, knowledge about web service topology is required, which can take the form of a web service schema. The web service schema maps the relationships between each of the web service resources and this allows a user to chain together a series web service requests, which will make it possible to answer the more complicated query. The ChEMBL web service schema diagram is displayed in Figure 2. Each of the oval shapes in Figure 2 represents a ChEMBL web service resource and the arrows between them indicates that resources share a common attribute. For example the Activity and Assay resources both contain the assay_chembl_id attribute. This means additional assay details, such as the assay_description, can be retrieved for activity data sets by making calls to the Assay resource. The direction of the arrow represents a foreign key constraint, found on the underlying relational database, for example the assay_chembl_id attribute is the unique identifier in the Assay resource, but occurs multiple times in the Activity resource. Using the relationships mapped out in the web service schema in collaboration with the online documentation makes it possible to answer complex queries using ChEMBL data.

### Data filtering and sorting

**Figure 3.**
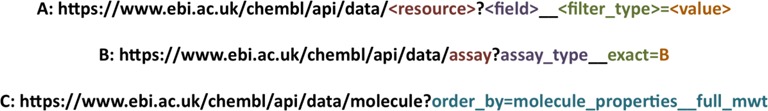
ChEMBL web service filtering and sorting examples. **A**: Example arguments for web service filter query (note the double underscore between <field> and <filter_type>). **B**: Example web service query returning binding (‘B’) assays. **C:** Example web service ordering query, which returns molecules ordered by molecular weight in ascending order. The ordering can be changed to descending order by placing a minus sign before the field name, e.g. ‘order_by = -molecule_properties__full_mwt’.

The ChEMBL web services allow for the retrieval of all entries for a specific resource. It is also possible to apply filters to searches using URL friendly query language built on top of the Django QuerySet API (https://docs.djangoproject.com/en/1.5/ref/models/querysets/). The format for submitting a filtered search is presented in Figure 3A, where the variables are the <resource>, <field>, <filter_type> and <value>. Based on this format, the URL displayed in Figure 3B will return Assays that have a ‘B’ assay_type. It is possible to apply multiple filters on a particular resource by separating multiple filter arguments with an ampersand character. To help optimize the filtering process additional filter_types are available and these are described in Table 2. It is also possible to order how results are returned to the user by using an ‘order_by = <field>’ argument. This is demonstrated in Figure 3C, where the molecule search results are returned ordered in ascending order based on the molecules full_mwt property.

**Table 2.** Example filter types that can be used in ChEMBL web services

Example	Description	Filter types
https://www.ebi.ac.uk/chembl/api/data/assay?assay_type__exact=B	Exact match with query	exact (iexact)
https://www.ebi.ac.uk/chembl/api/data/assay?description__icontains=toxicity	Wild card search with query	contains (icontains)
https://www.ebi.ac.uk/chembl/api/data/target?pref_name__istartswith=serotonin	Starts with query	startswith (istartswith)
https://www.ebi.ac.uk/chembl/api/data/cell_line?cell_source_tissue__iendswith=carcinoma	Ends with query	endswith (iendswith)
https://www.ebi.ac.uk/chembl/api/data/target?pref_name__iregex=(cdk1|cdk2)	Regular expression query	regex (iregex)
https://www.ebi.ac.uk/chembl/api/data/molecule?molecule_properties__full_mwt__gte=100	Greater than (or equal)	gt (gte)
https://www.ebi.ac.uk/chembl/api/data/molecule?molecule_properties__alogp__lte=5	Less than (or equal)	lt (lte)
https://www.ebi.ac.uk/chembl/api/data/molecule?molecule_properties__full_mwt__range=200,500	Within a range of values	range
https://www.ebi.ac.uk/chembl/api/data/molecule?molecule_chembl_id__in=CHEMBL25,CHEMBL941,CHEMBL1000	Appears within list of query values	in
https://www.ebi.ac.uk/chembl/api/data/assay?assay_tissue__isnull=false	Field is null	isnull

The ‘i’ versions of filter types, e.g. iexact, represent case insensitive forms. The ‘gt’ and ‘lt’ filter_type examples demonstrate how to access a field within a nested block. In these cases the full_mwt and alogp fields are contained within a ‘molecule_properties’ block. To access the fields contained within this section, the field name is double underscore prepended with the outer block name, e.g. ‘molecule_properties__full_mwt’.

### Chemical searching and representation

The Substructure and Similarity resources extend the previously defined data model, by providing users with additional chemical search functionality. Using these resources it is possible to search the molecules in the ChEMBL database, that have a molfile (9), using the Simplified Molecular Input Line Entry System (SMILES) representation (10) of another molecule. It is also possible to search for molecules within the ChEMBL database, using the molecule ChEMBL_ID or InChI key. This enables users to quickly define the chemical space for a given molecule within the ChEMBL database, based on ChEMBL_ID or commonly used chemical representations. Example substructure and similarity queries are provided in Supplementary Table S2.

The Image resource provides a graphical representation of a ChEMBL molecule and can be searched with either ChEMBL_ID or InChI key. Instead of accepting pagination and filtering arguments, the Image resource accepts methods that allow user to select format (png, svg and json), structure rendering engine (RDKit or Indigo, http://www.rdkit.org, http://lifescience.opensource.epam.com/indigo), reset molfile coordinates and output image size. Supplementary Table S3 provides more details on the arguments accepted by the Image resource.

### Web service client example

**Figure 4.**
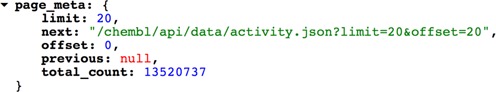
ChEMBL web service page_meta section from a request to https://www.ebi.ac.uk/chembl/api/data/activity.json.

The complex query, which returned *protein targets that interact with drugs classified as being used in the treatment of diabetes*, can be answered by making a series of requests to the ChEMBL web services. An example workflow that could be used to answer this question is outlined in Table 3. When reviewing the steps in Table 3, it should be noted that additional programmatic steps are required to parse and store the data being returned by the web service requests. To return requests for large data sets, e.g. exceeding 13 million activity values, the ChEMBL web services have implemented a result-set paging mechanism. Information regarding the number of hits, the number of pages, number of hits per page and the URLs to the next and previous result-set page are captured in page_meta section. An example of the page_meta section is provided in Figure 4. Parsing the data stored in the page_meta allows a user to quickly retrieve and process a large result-set in batches.

**Table 3.** Example ChEMBL web service calls, which would return protein targets that interact with drugs classified as being used in the treatment of diabetes

Step	Description	URL
1	Return the distinct set of molecules that match ATC codes starting with ‘A10’	https://www.ebi.ac.uk/chembl/api/data/molecule?atc_classifications__level5__startswith=A10
2	Return the distinct set of targets from the activities resource for previously matched molecules, where the pChEMBL value is greater than or equal to 6. For each molecule returned in Step 1, the following example URL will be requested, changing the molecule_chembl_id each time.	https://www.ebi.ac.uk/chembl/api/data/activity?molecule_chembl_id=CHEMBL429910&pchembl_value__gte=6
3	Return additional target data e.g. name, organism, target type and accessions. For each target returned in Step 2, the following example URL will be requested, changing the target chembl_id each time.	https://www.ebi.ac.uk/chembl/api/data/target/CHEMBL216

Additional processing of results are required at each stage to generate final results.

**Figure 5.**
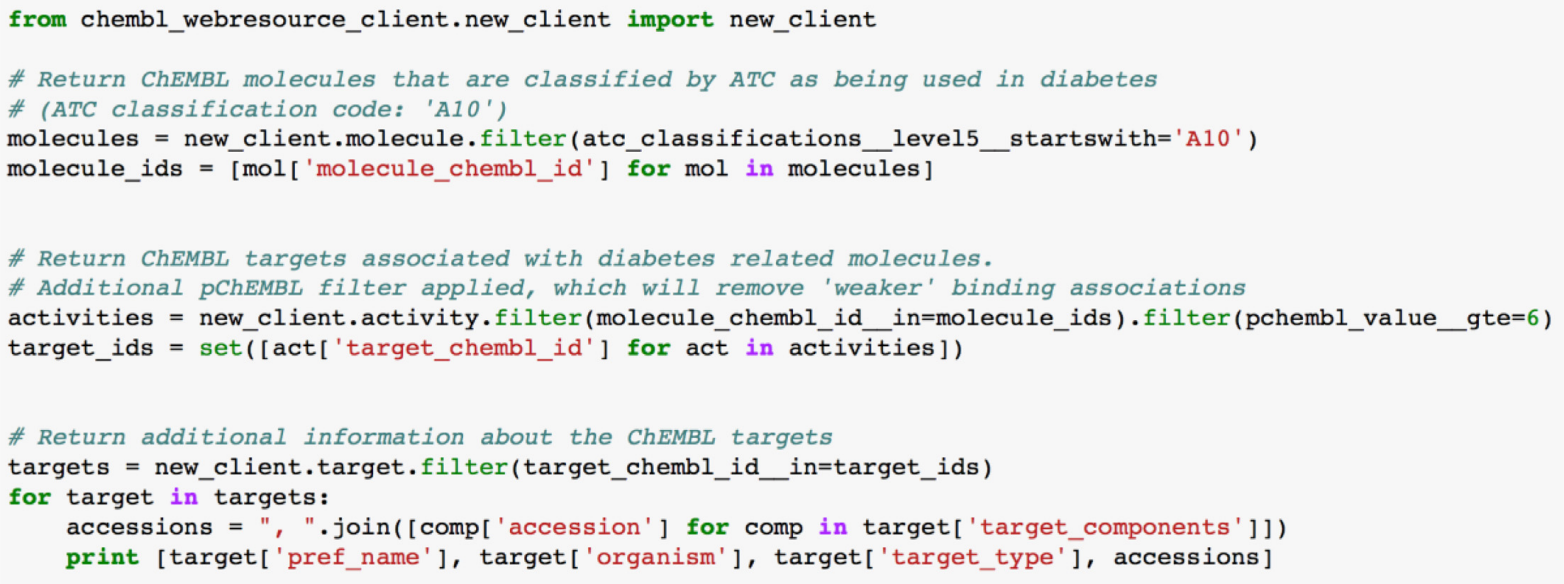
Example of using the ChEMBL web service client to return protein targets that interact with drugs classified as being used in the treatment diabetes.

Accessing the ChEMBL web services via the raw URLs allows users to easily integrate ChEMBL data into applications and retrieve data using command line tools such as, cURL (http://curl.haxx.se/). To minimize the required results parsing, a ChEMBL web resource client (https://github.com/chembl/chembl_webresource_client) has been created for the Python programming language. The client handles interaction with the HTTPS protocol and caches all results in the local file system for faster retrieval. Abstracting away all network-related tasks, the client provides the end user with a convenient interface, giving the impression of working with a local resource. The client code base is small, easy to install and its design is based on the Django QuerySet interface (https://docs.djangoproject.com/en/1.5/ref/models/querysets/). The client also implements lazy evaluation of results, which means it will only evaluate a request for data when a value is required. This approach reduces number of network requests and increases performance. The simplified version of workflow in Table 3 using the ChEMBL web service client is presented in Figure 5.

## TECHNICAL IMPLEMENTATION

**Figure 6.**
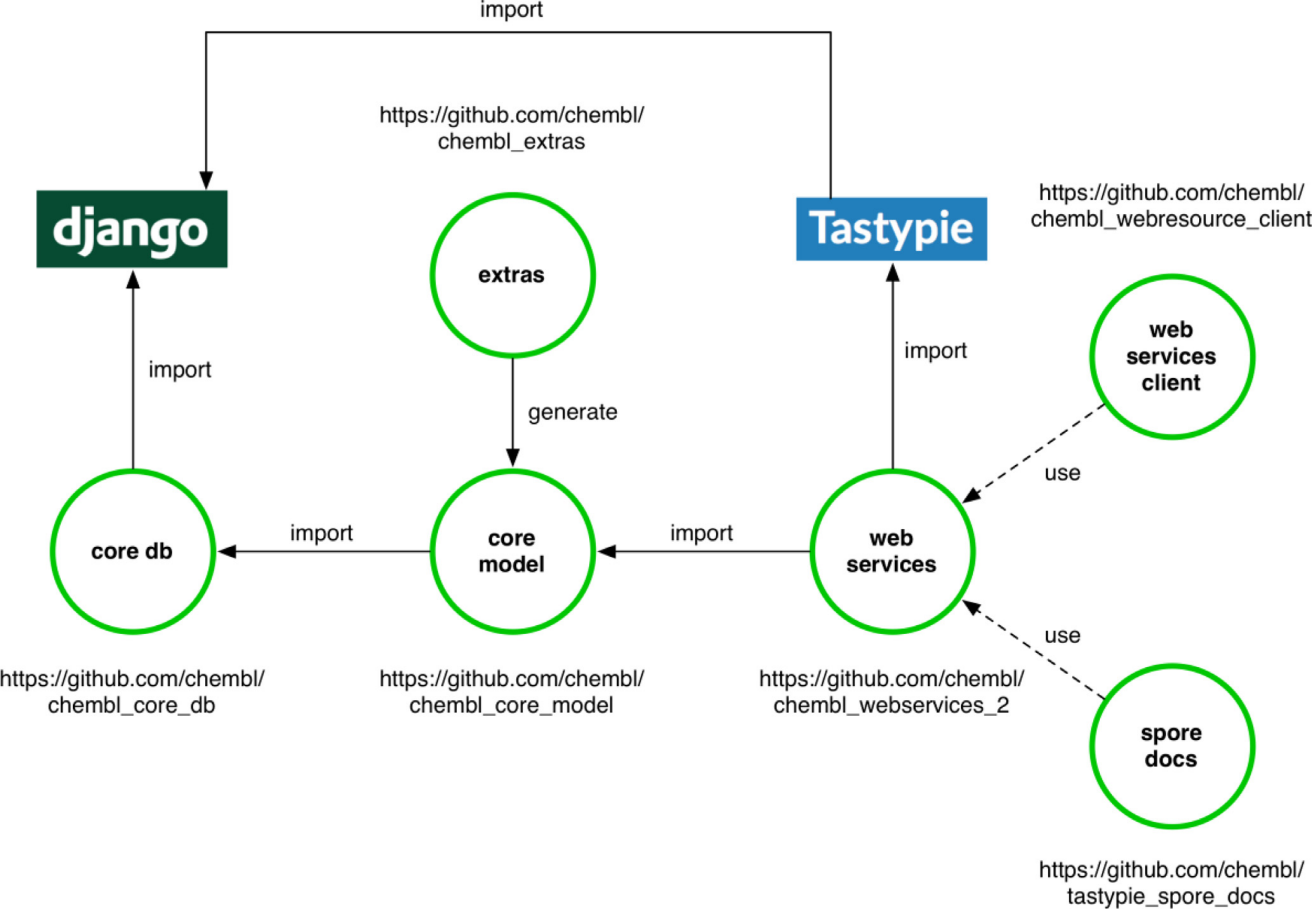
ChEMBL web service software components. The green circles correspond to python libraries developed by the ChEMBL group and are available on the ChEMBL GitHub site. Django (https://www.djangoproject.com/) and Tastypie (https://django-tastypie.readthedocs.org) are open source python libraries, which are core dependencies of the ChEMBL web services.

The ChEMBL web services are built on top of the modular software stack, consisting of many reusable components, which is illustrated in Figure 6. All of the components are written in Python programming language and are used within Django software framework (https://www.djangoproject.com/). The ‘chembl_core_model’ package (https://github.com/chembl/chembl_core_model) is responsible for providing the previously described ORM representation of the ChEMBL database. The ‘chembl_core_model’ package is generated automatically by inspection of the ChEMBL database using the ‘reverseEngineer’ command provided in the ‘chembl_extras’ package (https://github.com/chembl/chembl_extras). The automatically generated model is manually inspected before release to ensure any new data types and constraints in the underlying ChEMBL database are represented correctly in the ORM-based model.

Access between the ORM-based model and the backend ChEMBL database, which could be Oracle, MySQL, PostgreSQL or any other supported database engine, is provided through the ‘chembl_core_db’ package (https://github.com/chembl/chembl_core_db). To reduce load on the underlying database and improve performance a custom caching solution has been built on top of the standard Django cache backend interface (https://docs.djangoproject.com/en/1.5/topics/cache/#using-a-custom-cache-backend). The cache implementation uses MongoDB (https://www.mongodb.org/) as a data store. The decision to use MongoDB was based on the following factors. Firstly, the contents stored within a MongoDB will survive a server reboot, unlike memcached (http://www.memcached.org/) based solutions and certain caching mechanisms available in the underlying database (e.g. ORACLE CACHE Optimizer Hint). Secondly, MongoDB also supports data replication and load balancing, which was a very important requirement for the scalable production environment in which the ChEMBL web services run. The cache works by storing the data requests as a zlib compressed, base64-encoded pickle (https://docs.python.org/2/library/pickle.html) of QuerySet (https://docs.djangoproject.com/en/1.5/ref/models/querysets/) results. This process happens transparently and also overcomes the MongoDB maximum document size limitation, by chunking the objects into 16 MB blocks. A configurable timeout is set on every interaction with the MongoDB cache to ensure that the whole process will be faster than requesting the data directly from the database. Additional configurable parameters include compression level, chunk size and cache-key generation algorithm.

The ‘chembl_webservices_2’ package (https://github.com/chembl/chembl_webservices_2) is responsible for exposing the ChEMBL ORM model as a web service. The ‘chembl_webservices_2’ package captures the mapping between the web resource fields and the ORM classes, defined in the ‘chembl_core_model’ package. The ‘chembl_webservices_2’ package also uses the Tastypie API framework (https://django-tastypie.readthedocs.org/en/latest/), to handle all functionality associated with a RESTful interface, such as filtering, pagination, interaction with cache and error handling.

## CHEMISTRY AS A SERVICE

Access to the ChEMBL database *via* a web service can be seen as a ‘data-focused’ service, as it is responsible for retrieving data stored in the ChEMBL database. To assist with data processing, loading and curating, a requirement to build additional ‘cheminformatics-focused’ services was identified. Examples of desired routine operations provided by such a service include property calculations (e.g. molecular weight), conversion between various structure formats (e.g. molfile to SMILES), structure standardisation and extraction of chemical information from their graphical representation. Access to such services is normally provided through language specific chemical software libraries, e.g. RDKit and Chemistry Development Kit (CDK) (11). To remove the language dependency and the often non-trivial installation process, access to these services is provided via a web service. In line with the Service Oriented Architecture principles, this ‘*Chemistry As A Service’* approach facilitates the deployment of a single, centralized instance of the software and allows the underlying cheminformatics methods to be accessed and consumed by all languages, application frameworks and workflow tools that support access to RESTful web services. The service, ‘ChEMBL Beaker’ (https://github.com/chembl/chembl_beaker), is a light-weight application that wraps the functionality of the RDKit open source cheminformatics library (12). ChEMBL Beaker resources, which can be accessed by both GET and POST requests, are listed in Supplementary Table S4. ChEMBL Beaker has become an integral part of the new ChEMBL web services infrastructure, creating a convenient and powerful tandem with data-focused resources. Interactive documentation is also available for the ChEMBL Beaker services (https://www.ebi.ac.uk/chembl/api/utils/docs).

## USE CASE

### ChEMBL bioactivity data for a molecule extracted from an image

**Figure 7.**
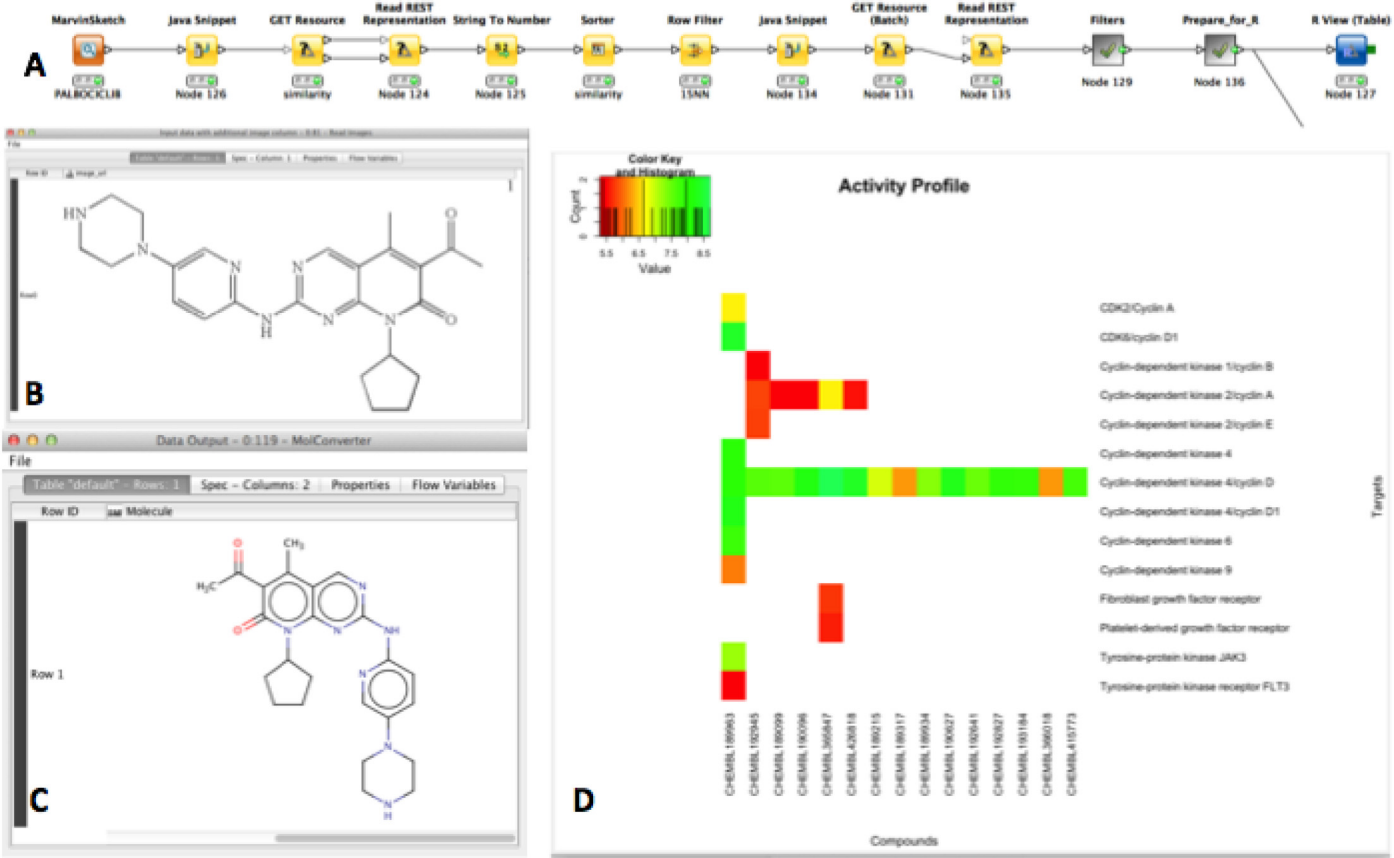
(**A**) KNIME workflow combining data and utility ChEMBL web service requests. (**B**) The image of the drug palbociclib is extracted from a patent document and then converted to the corresponding 2D structure via a Beaker call - shown in (**C**). The structure is then used as a query in a similarity search web service call against the ChEMBL database. Finally, all bioactivities for the drug and its close analogues are retrieved via a third web service call. (**D**) The resulting bioactivities and filtered and plotted in a heat map across compounds and their corresponding biological targets.

The ChEMBL web services can be called via any programming language or workflow tool, such as Taverna (13), KNIME (14) or Pipeline Pilot (http://www.accelrys.com/products/pipeline-pilot). The latter two, in particular, have been increasingly adopted by the computational and medicinal chemistry community, mainly due to their ease of use and the number of chemistry and cheminformatics extensions available. This use case was implemented here as a KNIME (version 2.11) workflow, summarized in Figure 7. The workflow takes advantage of the KREST node extensions (http://tech.knime.org/book/krest-rest-nodes-for-knime-trusted-extension), which integrate RESTful functionality within KNIME. As the first step of the workflow, the user can manually sketch a query structure as input; here the structure of the recently approved drug palbociclib (CHEMBL189963) was used. Alternatively, the user can provide an image (PNG, JPEG or TIFF format), of a structure, which is then converted to the corresponding 2D structure by calling the image2ctab Beaker web service. The query is then submitted to the similarity search web service and the hits are retrieved and sorted by similarity in a KNIME table. The 15 closest analogues are used as input for the activity web service call, which in turn retrieves all bioactivities in ChEMBL for these molecules. Finally, after the bioactivities are filtered and summarized, the data are presented as an activity profile heat map chart, produced by R (Figure 7D).

The example workflow presented above illustrates the power of web services tightly coupled with the data analysis capabilities of KNIME. ChEMBL data are readily retrieved within KNIME without the need of a local database or a chemical database cartridge. Additional cheminformatics functionality (e.g. image to structure conversion) is available seamlessly without the need to install the necessary tools locally, e.g. OSRA (http://cactus.nci.nih.gov/osra/). Finally, the data can be easily further processed, manipulated and visualized in KNIME, which provides additional integration with other established data mining tools such as Python and R. Importantly, this data and analysis combination of functionalities is freely available to everyone. The example KNIME workflow can be downloaded from https://www.ebi.ac.uk/chembl/extra/ChEMBL_WSv2_public.zip.

## DISTRIBUTION OF CHEMBL WEB SERVICES

The ChEMBL web services are hosted on EMBL-EBI infrastructure and are made freely accessible to all users over a secure HTTPS connection. Users working in intellectual property sensitive settings may be restricted from accessing publicly hosted services, even when using a HTTPS connection. To ensure all users are able to access the ChEMBL web services a number of measures have been taken. Firstly, we have made the code base for the ChEMBL web services available on GitHub (https://github.com/chembl) under the standard OSI-approved open source Apache 2.0 License (https://www.apache.org/licenses/LICENSE-2.0). For convenience, the software is divided into small reusable packages (described in technical implementation section), which have also been registered into Python Package Index (https://pypi.python.org/pypi). Access to the source code will provide an acceptable solution for many users looking to access the ChEMBL web services behind a cooperate firewall. This also allows software developers to easily reuse and extend the functionality of the ChEMBL web services. The local installation of the ChEMBL web services will require additional dependencies, most importantly the ChEMBL relational database with a supported chemical cartridge installed. This is a non-trivial setup and may be beyond the technical capabilities of some users. To assist users in this situation, an alternative approach to getting a local installation of the ChEMBL web services is to use myChEMBL (https://github.com/chembl/mychembl), which provides users with a local secure version of the ChEMBL web services.

## DISCUSSION

The ChEMBL web services have seen a significant enhancement in the data and functionality they offer. The first release of the updated services can be considered a foundation release, on to which new resources and search-based services will be built. The addition of new data resources will be tightly coupled to the ChEMBL release, where the underlying data model is often extended. As the ChEMBL web services are built on top of an ORM-based software model, which reflects the underlying relational model of the ChEMBL database, exposing new data resources is a very straightforward process. With regard to the addition of new search-based services, this will very much depend on the feedback from users of the service and the availability and ease of integration of new technologies. Follow on extensions to this project may include the addition of a BLAST (15) search resource that will allow users to run sequence-based searches against protein targets and biotherapeutics. We also intend to integrate SOLR (https://lucene.apache.org/solr/) searching capabilities, as an extension of the existing filtering mechanism. By using SOLR indexes as an optional backend to the ChEMBL web services, users will benefit from significantly faster query execution times and the ability to write complex filters using Lucene (https://lucene.apache.org/) based query syntax.

The primary goal of the ChEMBL web services is to deliver ChEMBL data and services in a machine-readable format that is easy for an application to consume and process further. Therefore, we consider these services to be a platform on which more complex tools and applications can be built or integrated. For example, by supporting cross-origin resource sharing (CORS) and JSONP, the ChEMBL data can easily be integrated with web-based applications. The availability of the web services client makes the process of integration even easier for developers using the Python programming language. The provision of additional language specific clients is a desired extension to the project, but we would like to encourage our users to assist in this endeavour by contributing their own efforts to the community.

Finally, it is hoped that the recent changes that have been made to ChEMBL web services will improve the accessibility of the ChEMBL data to both researchers and software developers working in the field of drug discovery.

## SUPPLEMENTARY DATA

Supplementary Data are available at NAR Online.

SUPPLEMENTARY DATA
