# Supplementary material for: ChEMBL web services: streamlining access to drug discovery data and utilities
Source: Nucleic Acids Res. 2015 Apr 16;43(Web Server issue):W612–20. doi: 10.1093/nar/gkv352 (PMC4489243; doi:10.1093/nar/gkv352)
Supplement: SUPPLEMENTARY DATA [file supp_43_W1_W612__index.html]

ChEMBL web services: streamlining access to drug discovery data and utilities — SUPPLEMENTARY DATA 

# ChEMBL web services: streamlining access to drug discovery data and utilities

## SUPPLEMENTARY DATA

**Files in this Data Supplement:**

- SUPPLEMENTARY DATA
- SUPPLEMENTARY DATA
- SUPPLEMENTARY DATA
- SUPPLEMENTARY DATA
