# Supplementary material for: ChEMBL web services: streamlining access to drug discovery data and utilities
Source: Nucleic Acids Res. 2015 Apr 16;43(Web Server issue):W612–20. doi: 10.1093/nar/gkv352 (PMC4489243; doi:10.1093/nar/gkv352)
Supplement: SUPPLEMENTARY DATA [file supp_gkv352_nar-00476-web-b-2015-File012.docx]

| **Feature** | **Original ChEMBL Web Services** | **Updated ChEMBL Web Services** |
| --- | --- | --- |
| Base URL | https://www.ebi.ac.uk/chemblws | https://www.ebi.ac.uk/chembl/api/data |
| Number of resources | 5 | 18 |
| Pagination | No | Yes |
| Filtering | No | Yes |
| Ordering | No | Yes |
| Raster Images | Yes | Yes |
| Vector Images | No | Yes |
| [JSONP support](https://en.wikipedia.org/wiki/JSONP) | Yes | Yes |
| [CORS support](https://en.wikipedia.org/wiki/Cross-origin_resource_sharing) | Yes | Yes |
| Online Documentation | [Yes](https://www.ebi.ac.uk/chemblws/docs) | [Yes](https://www.ebi.ac.uk/chembl/api/data/docs) |
| Python client library | Yes | Yes |
| Available REST verbs | GET, POST | GET, POST |
| Support for SMILES in GET | Partial | Full |

Supplementary Table 1. Comparison between original ChEMBL web services release and the updated ChEMBL web services.
