# Supplementary material for: ChEMBL web services: streamlining access to drug discovery data and utilities
Source: Nucleic Acids Res. 2015 Apr 16;43(Web Server issue):W612–20. doi: 10.1093/nar/gkv352 (PMC4489243; doi:10.1093/nar/gkv352)
Supplement: SUPPLEMENTARY DATA [file supp_gkv352_nar-00476-web-b-2015-File013.docx]

| **Chemical Search Description** | **Example Query URL** |
| --- | --- |
| Substructure search for against ChEMBL using aspirin SMILES string | https://www.ebi.ac.uk/chembl/api/data/substructure/CC(=O)Oc1ccccc1C(=O)O |
| Substructure search for against ChEMBL using aspirin CHEMBL_ID | https://www.ebi.ac.uk/chembl/api/data/substructure/CHEMBL25 |
| Substructure search for against ChEMBL using aspirin InChI Key | https://www.ebi.ac.uk/chembl/api/data/substructure/BSYNRYMUTXBXSQ-UHFFFAOYSA-N |
| Similarity (80% cut off) search for against ChEMBL using aspirin SMILES string | https://www.ebi.ac.uk/chembl/api/data/similarity/CC(=O)Oc1ccccc1C(=O)O/80 |
| Similarity (80% cut off) search for against ChEMBL using aspirin CHEMBL_ID | https://www.ebi.ac.uk/chembl/api/data/similarity/CHEMBL25/80 |
| Similarity (80% cut off) search for against ChEMBL using aspirin InChI Key | https://www.ebi.ac.uk/chembl/api/data/similarity/BSYNRYMUTXBXSQ-UHFFFAOYSA-N/80 |

Supplementary Table 2. Example ChEMBL web service substructure and similarity chemical search queries
