# Supplementary material for: ChEMBL web services: streamlining access to drug discovery data and utilities
Source: Nucleic Acids Res. 2015 Apr 16;43(Web Server issue):W612–20. doi: 10.1093/nar/gkv352 (PMC4489243; doi:10.1093/nar/gkv352)
Supplement: SUPPLEMENTARY DATA [file supp_gkv352_nar-00476-web-b-2015-File014.docx]

| **Argument** | **Description** | **Allowed Values** | **Default value** | **Example URL** |
| --- | --- | --- | --- | --- |
| format | Image format | png, svg and json | png | https://www.ebi.ac.uk/chembl/api/data/image/CHEMBL25?format=svg |
| dimensions | Size of image in pixels | 1-500 | 500 | https://www.ebi.ac.uk/chembl/api/data/image/CHEMBL25?dimensions=200 |
| ignoreCoords | Choose to use or ignore coordinates in ChEMBL molfiles | 1 or 0 | 0 (Use ChEMBL molfile coordinates) | https://www.ebi.ac.uk/chembl/api/data/image/CHEMBL25?ignoreCoords=1 |
| engine | Chemical toolkit used to generate image | rdkit or indigo | rdkit | https://www.ebi.ac.uk/chembl/api/data/image/CHEMBL25?engine=indigo |

Supplementary Table 3. Additional arguments that can be used when making a request to the ChEMBL web service Image resource.
