# Supplementary material for: ChEMBL web services: streamlining access to drug discovery data and utilities
Source: Nucleic Acids Res. 2015 Apr 16;43(Web Server issue):W612–20. doi: 10.1093/nar/gkv352 (PMC4489243; doi:10.1093/nar/gkv352)
Supplement: SUPPLEMENTARY DATA [file supp_gkv352_nar-00476-web-b-2015-File015.docx]

| **Name** | **Description** | **Example** |
| --- | --- | --- |
| break_bonds | Break bonds to Group I or II metals | curl --data-binary @a.sdf https://www.ebi.ac.uk/chembl/api/utils/break_bonds |
| canonicalizeSmiles | Converts SMILES to canonical form | curl -d 'O=C(Oc1ccccc1C(=O)O)C' https://www.ebi.ac.uk/chembl/api/utils/canonicalizeSmiles |
| ctab23D | Computes 3D coordinates of molecule in MDL molfile | curl --data-binary @a.sdf https://www.ebi.ac.uk/chembl/api/utils/ctab23D |
| ctab2image | Converts MDL molfile to molecule png image | curl --data-binary @a.sdf <https://www.ebi.ac.uk/chembl/api/utils/ctab2image> > a.png |
| ctab2inchi | Computes a standard InChI from MDL molfile | curl --data-binary @a.sdf https://www.ebi.ac.uk/chembl/api/utils/ctab2inchi |
| ctab2json | Converts MDL molfile to graphical representation encoded in JSON format | curl --data-binary @a.sdf https://www.ebi.ac.uk/chembl/api/utils/ctab2json |
| ctab2smiles | Converts MDL molfile to SMILES notation. | curl --data-binary @a.sdf https://www.ebi.ac.uk/chembl/api/utils/ctab2smiles |
| ctab2svg | Converts MDL molfile to molecule svg graphic | curl --data-binary @a.sdf <https://www.ebi.ac.uk/chembl/api/utils/ctab2svg> > a.svg |
| descriptors | Calculates set of chemical molecule descriptors from MDL molfile | curl --data-binary @a.sdf https://www.ebi.ac.uk/chembl/api/utils/descriptors |
| getNumAtoms | Returns number of atoms in molecule from MDL molfile | curl --data-binary @a.sdf https://www.ebi.ac.uk/chembl/api/utils/getNumAtoms |
| image2ctab | Performs optical structure recognition on molecule image and returns MDL molfile | curl --data-binary @mol.png https://www.ebi.ac.uk/chembl/api/utils/image2ctab |
| image2smiles | Performs optical structure recognition on molecule image and returns SMILES | curl --data-binary @mol.png https://www.ebi.ac.uk/chembl/api/utils/image2smiles |
| inchi2ctab | Converts InChI to MDL molfile format | curl -d 'InChI=1S/C9H8O4/c1-6(10)13-8-5-3-2-4-7(8)9(11)12/h2-5H,1H3,(H,11,12)' https://www.ebi.ac.uk/chembl/api/utils/inchi2ctab |
| inchi2inchiKey | Computes InChI key from InChI | curl -d 'InChI=1S/C9H8O4/c1-6(10)13-8-5-3-2-4-7(8)9(11)12/h2-5H,1H3,(H,11,12)' https://www.ebi.ac.uk/chembl/api/utils/inchi2inchiKey |
| kekulize | Brings a molecule from MDL molfie to Kekulé form | curl --data-binary @a.sdf https://www.ebi.ac.uk/chembl/api/utils/kekulize |
| logP | Computes Partition coefficient of the molecule in MDL molfile. | curl --data-binary @a.sdf https://www.ebi.ac.uk/chembl/api/utils/logP |
| mcs | Computes Maximum Common Substructure of two molecules from MDL molfile | curl --data-binary @a.sdf https://www.ebi.ac.uk/chembl/api/utils/mcs |
| molWt | Calculates molecular weight of the molecule in MDL molfile | curl --data-binary @a.sdf https://www.ebi.ac.uk/chembl/api/utils/molWt |
| neutralise | Neutralize charges by adding/removing protons | curl --data-binary @a.sdf https://www.ebi.ac.uk/chembl/api/utils/neutralise |
| rules | Apply standardization rules (https://www.ebi.ac.uk/chembl/extra/francis/standardiser/Rule_application_strategy.html) | curl --data-binary @a.sdf https://www.ebi.ac.uk/chembl/api/utils/rules |
| sdf2fps | Calculates fingerprints of structure from MDL molfile | curl --data-binary @a.sdf https://www.ebi.ac.uk/chembl/api/utils/sdf2fps |
| smiles23D | Computes 3D coordinates of molecule represented as SMILES | curl -d 'CCN' https://www.ebi.ac.uk/chembl/api/utils/smiles23D |
| smiles2ctab | Converts molecule format from SMILES to MDL molfile | curl -d 'CCN' https://www.ebi.ac.uk/chembl/api/utils/smiles2ctab |
| smiles2image | Renders png image of molecule structure from SMILES representation | curl -d 'CCN' https://www.ebi.ac.uk/chembl/api/utils/smiles2image > mol.png |
| smiles2json | Converts SMILES to graphical representation encoded in JSON format | curl -d 'CCN' https://www.ebi.ac.uk/chembl/api/utils/smiles2json |
| smiles2svg | Renders svg graphic of molecule structure from SMILES representation | curl -d 'CCN' https://www.ebi.ac.uk/chembl/api/utils/smiles2svg |
| standardise | Get standardised parent | curl --data-binary @a.sdf https://www.ebi.ac.uk/chembl/api/utils/standardise |
| status | Web service status | curl https://www.ebi.ac.uk/chembl/api/utils/status |
| tpsa | Compues Polar Surface Area for molecule in MDL molfile | curl --data-binary @a.sdf https://www.ebi.ac.uk/chembl/api/utils/tpsa |
| unsalt | Discard any salt/solvate components | curl --data-binary @a.sdf https://www.ebi.ac.uk/chembl/api/utils/unsalt |

Supplementary Table 4. ChEMBL Beaker web service resources.
